# Modeling the Behavior of Red Blood Cells within the Caudal Vein Plexus of Zebrafish

**DOI:** 10.3389/fphys.2016.00455

**Published:** 2016-10-07

**Authors:** Tijana R. Djukic, Swapna Karthik, Igor Saveljic, Valentin Djonov, Nenad Filipovic

**Affiliations:** ^1^Research and Development Center for Bioengineering, BioIRCKragujevac, Serbia; ^2^Faculty of Mechanical Engineering, University of KragujevacKragujevac, Serbia; ^3^Topographic and Clinical Anatomy, Institute of Anatomy, University of BernBern, Switzerland; ^4^Graduate School for Cellular and Biomedical Sciences, University of BernBern, Switzerland; ^5^Harvard School of Public Health, Harvard UniversityBoston, MA, USA

**Keywords:** mathematical modeling, blood flow, deformable objects, solid-fluid interation, comparison with experimental data, caudal vein plexus, zebrafish embryo

## Abstract

Due to the important biological role of red blood cells (RBCs) in vertebrates, the analysis of reshaping and dynamics of RBCs motion is a critical issue in physiology and biomechanics. In this paper the behavior of RBCs within the immature capillary plexus during embryonic development of zebrafish has been analyzed. Relying on the fact that zebrafish embryos are small and optically transparent, it is possible to image the blood flow. In this way the anatomy of blood vessels is monitored along with the circulation throughout their development. Numerical simulations were performed using a specific numerical model that combines fluid flow simulation, modeling of the interaction of individual RBCs immersed in blood plasma with the surrounding fluid and modeling the deformation of individual cells. The results of numerical simulations are in accordance with the *in vivo* observed region of interest within the caudal vein plexus of the zebrafish embryo. Good agreement of results demonstrates the capabilities of the developed numerical model to predict and analyze the motion and deformation of RBCs in complex geometries. The proposed model (methodology) will help to elucidate different rheological and hematological related pathologies and finally to design better treatment strategies.

## Introduction

The process of development of an embryo and subsequent functioning of the cardiovascular system is important for the explanation of many phenomena occuring within this system. Vertebrates have a functional vasculature, with a heart that pumps blood and blood vessels that have a clearly defined endothelium. However, observation of the blood vessels in living embryos is difficult either because the embryos are developing within the uterus of the mother or because they are not transparent enough. A fish species called teleostei, more precisely a subspecies called zebrafish (in Latin *Danio rerio*), has great advantages over other species for studying vascular development. First of all, the dimension of zebrafish is very small, since an adult zebrafish can grow to 4 cm on average. Zebrafish are very fertile and lay a large number of eggs. The reproduction is external, outside the mother's body. Optical analysis of this species is also very easy. Due to its small dimensions, the embryos can survive with only a small amount of oxygen that they receive by passive diffusion. All the interior organs, such as eyes, brain, heart, inner ear are developed within the first 3 days post fertilization. The cardiovascular system is one of the first systems that is formed during the embryonic development of zebrafish. After only 1 day post fertilization, the zebrafish is sufficiently developed for the blood to start circulating. Detailed explanation of the formation of all major blood vessels in zebrafish can be found in the literature (Isogai et al., 2001; Ellertsdóttir et al., 2010). Even though there are certain variations in details of the anatomy of the cardiovascular system of this fish, the basics of the system are similar to other vertebrates. Because of all the mentioned advantages, many papers in the literature analyze many aspects of development of the cardiovascular system of zebrafish, and present numerous genetic analysis of mutation of diverse genes (Stainier et al., 1995; Weinstein et al., 1995). The zebrafish species has also been used for many diverse investigations, e.g., as a model to study angiogenesis (Chávez et al., 2016) or to examine the pathophysiology of myofibrillogenesis and muscular dystrophies (Raeker et al., 2014) etc. The cardiac and metabolic physiology of zebrafish was analyzed in the literature (Gore and Burggren, 2012), as well as development of the zebrafish heart *in vivo* (Hou et al., 2014).

There are also numerous studies that study the formation of erythrocytes in vertebrates, as well as in teleostei (Swaen and Brachet, 1899; Strawinski, 1949; Vernier, 1969). The process of generating blood cells is known as hematopoiesis. Zebrafish erythropoiesis begins in the mesodermal layer during embryonic development (Kulkeaw and Sugiyama, 2012). Zebrafish hematopoiesis undergoes two waves: primitive and definitive. Between 12 and 24 h post fertilization, primitive hematopoiesis starts in the intermediate cell mass (ICM), located between the somites and yolk sac. During this primitive wave, erythrocytes and macrophages are produced. By 24 h post fertilization, primitive erythrocytes enter circulation and mature. After maturation the erythrocytes retain the nucleus, elliptical shape and express hemoglobin (de Jong and Zon, 2005; Li et al., 2014).

The definitive hematopoiesis generates hematopoietic stem cells (HSCs) that differentiate into erythrocytes, lymphocytes, and platelets. It is also called adult hematopoiesis and it has the capability of self-renewal and produces all mature hematopoietic lineages (Falenta and Rodaway, 2011; Jin and Wen, 2011). In zebrafish, the *Runx1* transcription factor is crucial for HSCs formation, which is observed at early 24 h post fertilization. The *Gata-1*, transcription factor expression is vital for primitive hematopoiesis and it is found mainly in the lateral plate mesoderm that migrates medially during the formation of ICM. *Gata-1* positive cells are expressed during the differentiation of ICM to proerythroblasts (Li et al., 2014).

Using confocal microangiography (Weinstein et al., 1995) or green fluorescent protein (GFP, Motoike et al., 2000; Lawson and Weinstein, 2002), and relying on the fact that zebrafish are small and very transparent, it is possible to image the blood vessels throughout the entire depth of the zebrafish. This way the anatomy of the blood vessels is obtained and the flow of red blood cells (RBCs) through the blood vessel is monitored. Unlike some other techniques used for the analysis of the vasculature, the mentioned approach does not jeopardize in any way the fish and the obtained images are related to the fully active blood circulation.

Due to the important role of RBCs in vertebrates and human organisms, the analysis of the dynamics of motion of these cells separately is one of the most important problems in physiology and biomechanics. Many authors have analyzed the behavior of RBCs, both experimentally and theoretically. Behavior of synthetic capsules has been experimentally observed (Chang and Olbricht, 1993; Walter et al., 2000) and similar experiments were performed with RBCs (Gaehtgens et al., 1980; Pries and Secomb, 2011). Many authors have investigated the mechanical properties of erythrocytes, with a special focus on the characteristics of the cellular membrane, in the past century (Skalak, 1976; Hochmuth and Waugh, 1987), as well as in the past decade (Mukhopadhyay et al., 2002; Kuzman et al., 2004; Li et al., 2005). A theoretical model was used to simulate the motion of RBCs through capillaries with variable cross-sections, in order to predict the resistance of the vessel to the motion and deformation of RBCs in living microvessels (Secomb and Hsu, 1996). Recently, numerical simulations were performed on idealized arteriole-sized blood vessels and the influence of motion of RBCs on shear stress on the blood vessel walls was analyzed (Gambaruto, 2016). Secomb et al. (2007) performed experiments to analyze the behavior of human RBCs within a glass tube, that has a diameter smaller than the diameter of the RBC. This is similar to the conditions in human capillary blood vessels, which are also narrower than a single RBC. In this study, they observed the capability of an RBC to adapt to the changes in geometry during the flow and to change its shape significantly. Numerical simulations were also performed (Djukic and Filipovic, 2015) and compared with experimental results presented by Secomb et al. (2007). Through this comparison it was demonstrated that the proposed numerical model is capable of accurately predicting the change of shape that the RBC undergoes during its motion through a narrow glass tube. Most of the numerical models previously published in the literature consider only the motion of particles and RBCs in simpler geometrical conditions, where the high defomability of RBCs does not come to the fore. The goal of this paper is to model the motion of RBCs through a complex geometrical domain.

In this paper the behavior of RBCs within the caudal vein plexus during embryonic development of zebrafish is analyzed. The embryonic capillary plexus has its honeycomb-like appearance due to the aggregation of many transluminal intussusceptive pillars. The blood flow videos of observed phenomena in the capillary plexus regions were tracked *in vivo*, to isolate individual RBC movement. Subsequently, numerical simulations were performed in geometries and under conditions that are defined according to the experimental setup. The results are used to predict and analyze the motion and deformation of RBCs.

The paper is organized as follows: Section Materials and Methods describes the methods that were used to obtain experimental data and the numerical model that was used in numerical simulations. Section Results presents the results obtained in numerical simulations and the comparison with experimental data. Numerical methods are discussed in Section Discussion and Conclusion, including the conclusions about the significance of the presented results and applications of the proposed numerical model.

## Materials and methods

### Maintenance and *In vivo* imaging of zebrafish embryo experiments

Zebrafishes (*Danio rerio*) were maintained in a facility with the system water at 28.5°C with 14 h light: 10 h darkness circadian rhythm. An endothelial specific reporter transgenic line *Tg(fli1a:eGFP)*^*y7*^ was acquired from aquatic resource program (Children's Hospital, Boston, USA) and used for collecting *in vivo* experimental data. The transgenic embryos were attained from natural spawning with a 2:1 ratio (Female:Male) and staged according to the standard conditions (Westerfield, 2007). The embryos were kept in standard embryo medium (1X E3 medium) till 24 h post fertilization and screened for GFP expression. The dechorionated embryos were mounted on 0.5% of low melting point agarose gel containing E3 medium for imaging. The morphogenesis of the caudal vein plexus formation along with the blood flow videos was captured by fluorescence stereomicroscopy (Leica stereomicroscope M205FA, Leica microsystems, Switzerland). Still images were captured and blood flow was recorded as video files using a Leica camera (DFC365X) and software (Leica AF600). All the animal experiments were performed according to the guidelines of the Swiss animal welfare act. According to the Swiss government guidelines, experiments performed on zebrafish embryos aged less than 48 h of post fertilized embryos are exempted from the animal permission.

### Numerical model

The numerical model presented in this paper simulates fluid flow at the microscale level. The motion and deformation of individual RBCs is analyzed. These cells are immersed in blood plasma and interact with the surrounding fluid, i.e., blood plasma. Cells influence fluid flow and on the other hand, fluid causes the deformation of cells. In the sequel of this Section, details of the numerical model is described.

#### Fluid flow simulation

In this paper the Lattice Boltzmann (LB) method was used to simulate fluid flow. This method was successfully applied to modeling the motion of solid bodies through a fluid domain (Sun et al., 2003; Dupin et al., 2007; Wu and Shu, 2010), the motion of LDL (low-density lipoprotein) particles (Filipovic et al., 2014) and nanodrugs (Filipovic et al., 2012) through the bloodstream, as well as the motion of deformable circulating tumor cells through a microfluidic chip (Djukic et al., 2015). In the LB method, fluid is observed as a set of fictional particles that are located within a fixed Cartesian mesh and the dynamics of motion of these particles is studied through their mutual collisions and further propagation in the observed domain. Details of this method can be found in the literature (Malaspinas, 2009; Djukic, 2012).

Within the LB method, a special principle is applied for calculation of all physical quantities, such that all macroscopic quantities required for the simulation and all quantities obtained as the result of the simulation are defined in the so-called system of lattice units. This system represents all quantities in its dimensionless form, related to the defined lattice mesh. Hence it is necessary to determine the values of all parameters in dimensionless form before starting the simulation. Also, when the results are post-processed, it is necessary to tranfsorm dimensionless quantities back to the physical quantities. In order to perform this transformation, three relevant scale factors are calculated—scale factor for time, length, and density. Any other physical quantity can be expressed in terms of these three quantities, so these three scale factors are sufficient for all transformations. Additional details about this procedure can be found in the literature (Djukic, 2012).

The basic quantity in the LB method is the distribution function *f*, that is defined such that *f*(**x**, *t*) represents the probability of a particle to be located within an element in space *dx* around point **x**, in moment in time *t*, where **x** denotes the particle position vector. When the LB method is implemented, the equation that represents the entire numerical scheme is most commonly separated into two steps—a collision step and a propagation step. Two values of distribution function are defined—$f_{i}^{in}$ and $f_{i}^{out}$, and they represent values of the discretized distribution function before and after collision.

The mentioned steps can be described using following equations:

Collision step:

(1) $$\begin{array}{r} f_{i}^{out}\left(\text{x},t\right)=f_{i}^{in}\left(\text{x},t\right)-\frac{1}{\tau }\left(f_{i}^{in}\left(\text{x},t\right)-f_{i}^{\left(0\right)}\left(\rho ,\text{u}\right)\right)\\ +\left(1-\frac{1}{2\tau }\right){\text{F}}_{i} \end{array}$$

Propagation step:

(2) $$\begin{array}{r} f_{i}^{in}\left(\text{x}+\xi_{i},t+1\right)=f_{i}^{out}\left(\text{x},t\right) \end{array}$$

These two steps are repeated in a series of iterations, whereas each of these two steps must be applied to all particles, i.e., nodes of the mesh, before the next step starts.

**Figure 1:**
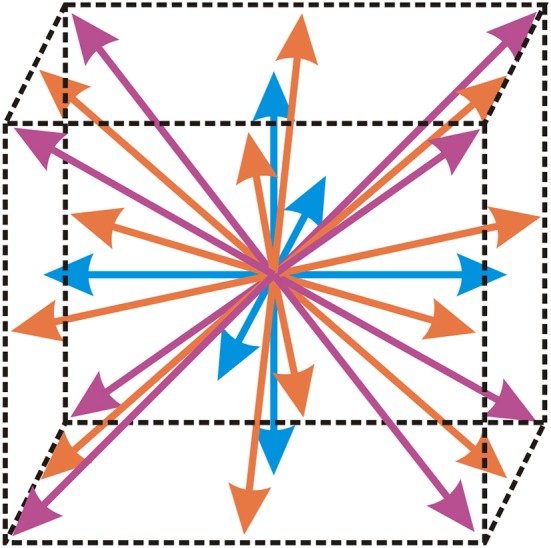
**Lattice structure D3Q27, that contains an overall of 27 different components of the distribution function**. Arrows denote the possible directions of motion of fictional fluid particles.

In the above equation, τ represents the relaxation time, **F**_*i*_ represents the discretized external force term, ρ represents the fluid density, **u** represents the fluid velocity, $f_{i}^{\left(0\right)}$ represents the equilibrium distribution function, **ξ**_*i*_ represent vectors defining the abscissae of the lattice structure and index *i* represents the component of the distribution function that is calculated. The abscissae for the three-dimensional isothermal flow of incompressible fluid used in this paper (denoted by D3Q27) are shown in Figure 1. This practically means that an overall of 27 different components (*i* = 1, …, 27) of the distribution function are calculated and these components are used to precisely define the possible directions of motion of fictional fluid particles in three-dimensional space.

The equilibrium distribution function is defined using the following equation:

(3) $$\begin{array}{r} f_{i}^{\left(0\right)}\left(\rho ,\text{u}\right)=\omega_{i}\rho \left(1+\frac{\xi_{i}\cdot \text{u}}{c_{s}^{2}}+\frac{1}{2c_{s}^{4}}{\left(\xi_{i}\cdot \text{u}\right)}^{2}-\frac{1}{2c_{s}^{2}}{\text{u}}^{2}\right) \end{array}$$

The discretized external force term is defined as:

(4) $$\begin{array}{r} {\text{F}}_{\text{i}}=\omega_{i}\rho \left(\frac{\xi_{i}-\text{u}}{c_{s}^{2}}+\frac{\left(\xi_{i}\cdot \text{u}\right)\xi_{i}}{2c_{s}^{4}}\right)\cdot \text{g}\left(\text{x},t\right) \end{array}$$

where **g** represents the external force field.

Macroscopic quantities necessary to describe the fluid flow at the macroscale level, such as density, pressure, velocity, are evaluated in terms of the calculated components of distribution function.

Density is calculated as:

(5) $$\begin{array}{r} \rho =\underset{i}{\sum }f_{i} \end{array}$$

The expression for velocity is given by:

(6) $$\begin{array}{r} \text{u}=\frac{1}{\rho }\underset{i}{\sum }\xi_{i}f_{i}=\text{u}-\frac{\text{g}}{2} \end{array}$$

Pressure is calculated in terms of the fluid density, in the considered node of the mesh, and this relation is given by:

(7) $$\begin{array}{r} p=c_{s}^{2}\rho \end{array}$$

where *c*_*s*_ represents a constant related to the LB method. For the D3Q27 lattice structure that is used in this paper, this constant is equal to $c_{s}^{2}=\frac{1}{3}$.

#### Modeling the deformation of the RBC

RBCs or erythrocytes are highly differentiated cells that contain a cellular membrane that surrounds the inner structure of the cell. In this study, an approximation is introduced, that assumes that the entire internal structure can be represented as an incompressible Newtonian fluid, because it is considered that the influence of the membrane is crucial for the deformation of the entire cell. The cellular membrane is composed of two layers of lipids and a thin skeleton of interconnected proteins (Evans and Skalak, 1980). Due to its structure, RBCs are highly deformable (Shiga et al., 1990; Maeda and Shiga, 1993). In this paper it is considered that the membrane of the RBC has negligible thickness and is interconnected with a predefined number of points. The discretization of the mesh that is used to model the membrane of the RBC is performed such that the entire membrane is divided on a defined number of triangles. During the simulation, the reaction force for every element and for every node of the triangular mesh is calculated. The resulting reaction force represents the resistance of a particular node to the defined external deformation.

There are four parameters that influence the behavior of the RBC, and these are: volume within the membrane, surface area of the membrane, surface strain of the membrane, and bending of the membrane. In this paper the membrane of the RBC is observed as a hyperelastic material, where the relationship between stress and strain can be defined using a strain energy density function. This also implies that the membrane of the RBC is incompressible and isotropic. Several hyperelastic material models have been developed and applied to model the surface strain of the RBC membrane, such as the Mooney-Rivlin and neo-Hookean material models (Ramanujan and Pozrikidis, 1998; Barthès-Biesel et al., 2002; Sui et al., 2008). However, Skalak et al. (1973) analyzed the mentioned models and came to the conclusion that these models are not able to simulate the behavior of RBCs accurately enough. They proposed a new material model for the membrane of the RBC which has been used in this study.

The strain energy density function in the Skalak material model of the deformable membrane is defined in terms of two invariants of the Cauchy-Green deformation tensor:

(8) $$\begin{array}{r} W^{S}=\frac{k_{s}}{12}\left(I_{1}^{\prime 2}+2I_{1}^{\prime }-2I_{2}^{\prime }\right)+\frac{k_{\alpha }}{12}I_{2}^{\prime 2} \end{array}$$

where $I_{1}^{\prime }$ and $I_{2}^{\prime }$ represent the modified invariants, as is proposed in the literature (Skalak et al., 1973). The following equations are used to define the modified invariants, in terms of the principal stretches λ_1_and λ_2_:

(9) $$\begin{array}{r} I_{1}^{\prime }=\lambda_{1}^{2}+\lambda_{2}^{2}-2 \end{array}$$

(10) $$\begin{array}{r} I_{2}^{\prime }=\lambda_{1}^{2}\lambda_{2}^{2}-1 \end{array}$$

The surface elastic shear modulus and area dilation modulus are denoted by *k*_*s*_ and *k*_α_, respectively. These two parameters are defined for the particular type of deformable body, in this case for the RBC.

Using the strain energy density function, it is possible to derive the equation that defines the stress-strain relationship:

(11) $$\begin{array}{l} \sigma_{ij}=\frac{1}{J}[\left(-\;\frac{k_{s}}{3}\;+\;\frac{k_{\alpha }}{3}I_{1}\left(I_{2}-1\right)\right)B_{ij}\\ \;\;-\left(-\frac{k_{s}}{3}\;+\;\frac{k_{\alpha }}{3}\left(I_{2}-1\right)\right)B_{ik}B_{kj}] \end{array}$$

where σ_*ij*_ represents the Cauchy stress tensor, *B* is the left Cauchy-Green deformation tensor, *J* is the determinant of the deformation gradient and *I*_1_ and *I*_2_ are tensor invariants expressed in terms of the Cauchy-Green deformation tensor.

In Equation (11), the tensor invariants are used, because this is more appropriate for the numerical calculation of the reaction force caused by the change of surface strain, which is calculated using the finite element method (Kojic et al., 2008). The remaining three components of the reaction force are calculated as proposed by Dupin et al. (2007).

The total reaction force of the deformable body caused by the deformation is calculated in each node, as a sum of forces calculated for all four types of deformation.

(12) $$\begin{array}{r} {\text{F}}_{i}\left(t\right)={\text{F}}_{i}^{S}+{\text{F}}_{i}^{V}+{\text{F}}_{i}^{A}+{\text{F}}_{i}^{B} \end{array}$$

In the above equation, ${\text{F}}_{\text{i}}^{\text{S}}$ represents the reaction force due to the surface strain of the membrane, ${\text{F}}_{\text{i}}^{\text{V}}$ represents the reaction force due to the change of volume within the membrane, ${\text{F}}_{\text{i}}^{\text{A}}$ represents the reaction force due to the surface area of the membrane and ${\text{F}}_{\text{i}}^{\text{B}}$ represents the reaction force due to the bending of the membrane.

#### Modeling the interaction between fluid and RBC

The immersed boundary method (IBM) presented by Peskin (1977) was used to model the interaction between the immersed RBC and surrounding fluid. This method was successfully applied for modeling the dynamics of motion of both rigid bodies (Feng and Michaelides, 2004; Wu and Shu, 2010; Djukic, 2012), and deformable objects (Krüger et al., 2011; Murayama et al., 2011; Djukic et al., 2015) immersed in fluid. The IBM observes the solid as an immersed object inside the fluid domain, where the boundary between the object and the surrounding fluid is assumed to be easily deformable, with high stiffness (Wu and Shu, 2010). The fluid affects the object, i.e., the membrane of the RBC through a force that deforms the membrane. Due to the deformation, an internal reaction force appears in the membrane and this force defines the effect of the RBC on the surrounding fluid. The fluid flow is simulated using the Navier-Stokes equations and the effect of the immersed object is introduced through an external force field in the fluid domain.

Since the discretization of the RBC membrane and fluid domain do not exactly overlap, the influence of various points from each mesh has to be taken into consideration when calculating quantities relevant for the simulation. Thus, an interpolation scheme has to be applied.

Interpolation is performed using the Dirac delta function, that is approximated as follows:

(13) $$\begin{array}{r} \delta \left(\text{x}-{\text{x}}_{B}\left(t\right)\right)=D_{ijk}\left({\text{x}}_{ijk}-{\text{x}}_{B}^{l}\right)\\ =\delta \left(x_{ijk}-X_{B}^{l}\right)\cdot \delta \left(y_{ijk}-Y_{B}^{l}\right)\cdot \delta \left(z_{ijk}-Z_{B}^{l}\right)\; \end{array}$$

where *D*_*ijk*_ is used to define the Dirac function at a specific point of the fluid domain, indexes *i*, *j* and *k* denote the currently considered point in the fluid mesh, ${\text{X}}_{B}^{l}\left(t\right)$, *l* = 1, 2, …, *G* are the coordinates of the *l*-th point of the mesh that is used to model the RBC membrane and *G* is the number of points in this mesh.

The value of function δ(*r*) is defined in the literature (Peskin, 1977):

(14) $$\delta (r)=\{\begin{array}{c} \frac{1}{4h}\;(1+\cos\;(\frac{\pi r}{2})),\;|r|\;\leq 2\\ 0,\;|r|\;>2 \end{array}$$

where *h* denotes the distance between two points of the fixed fluid mesh (in this case, since the LB method is used for fluid flow simulation, *h* = 1) and *r* is the distance between the considered points in the fluid and the mesh representing the membrane of the RBC.

As already mentioned, in fluid flow simulations the effect of the immersed object is introduced through an external force, that acts on the fluid surrounding the membrane. Due to the applied interpolation scheme, the reaction force at one point of the RBC membrane is transferred to several points of the fluid mesh. This force can be expressed as:

(15) $$\begin{array}{r} \text{g}\left({\text{x}}_{ij},t\right)=\overset{G}{\underset{l=1}{\sum }}{\text{F}}_{l}\left(t\right)D_{ij}\left({\text{x}}_{ij}-{\text{x}}_{B}^{l}\left(t\right)\right) \end{array}$$

where **F**_*l*_(*t*) is the force with which the *l*-th point of the mesh representing the membrane of the RBC opposes the effect of the surrounding fluid. Calculation of this force is explained in detail in Section Modeling the Deformation of the RBC.

The velocity of all points in the mesh representing the membrane of the RBC is interpolated over the surrounding points in fluid mesh, and it is calculated using the following equation:

(16) $$\begin{array}{r} {\text{u}}_{B}^{l}\left({\text{x}}_{B}^{l},t\right)=\underset{i,j}{\sum }\text{u}\left({\text{x}}_{ij},t\right)D_{ij}\left({\text{x}}_{ij}-{\text{x}}_{B}^{l}\right) \end{array}$$

Applying the Euler Forward method on Equation (16), the new positions of points in the mesh representing the membrane of the RBC are obtained:

(17) X t+ΔtBl=X tBl+uBlΔt

This way, the deformation of the RBC membrane at every time step is calculated. Using the obtained deformation, the reaction forces due to the new deformation of the membrane are calculated. These forces cause a change in the external force field in the fluid domain, thus causing an effect of the immersed object to the fluid. Fluid on the other hand, again causes new deformation of the RBC membrane. This process is repeated in each iteration, until the defined condition is satisfied or the defined number of iterations is reached.

## Results

**Figure 2:**
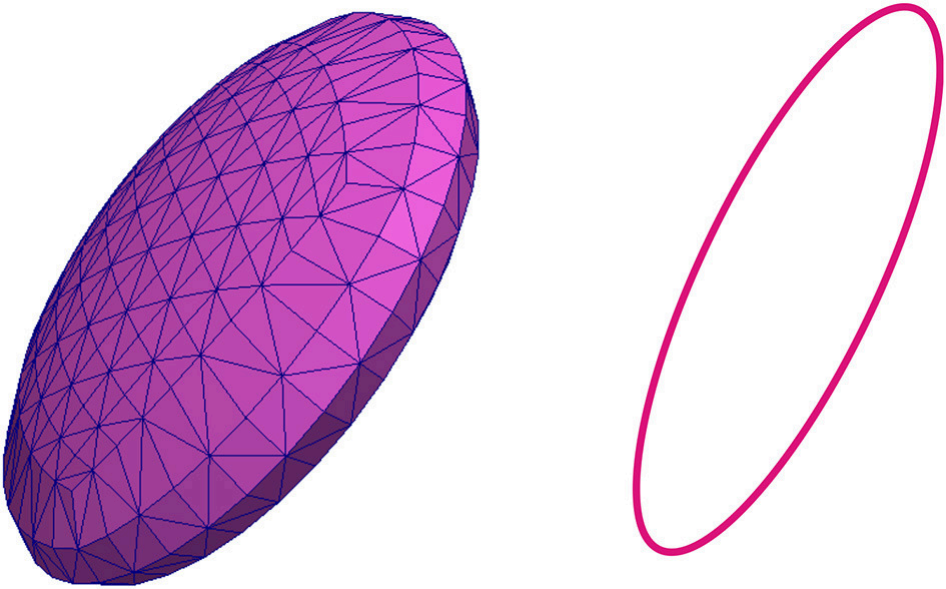
**Discretized model of the membrane representing the red blood cell that is used in numerical simulations (left) and the cross-section of the discretized model of the RBC along the plane that contains the center of gravity of the RBC (right)**.

In this paper it is considered that the RBC of the zebrafish has an elliptic shape, according to the previous investigations of the zebrafish cardiovascular system (Watkins et al., 2012). The model of the RBC membrane that is discretized into a triangular mesh and that is used in simulations presented in this paper is shown in Figure 2. The cross-section of RBC along the plane that is parallel to x0z plane and that contains the center of gravity of the RBC is also shown in Figure 2.

Because of the structure of RBCs and all the conditions to which they are exposed during its motion through the circulation, four types of resistance to deformation are defined. This was discussed in Section Modeling the Deformation of the RBC. Values of the parameters that define these reactions to deformation have been measured in the literature (Dao et al., 2003; Bagchi et al., 2005). For modeling the behavior of RBCs in this paper, the values are taken to be equal to the ones proposed by Dupin et al. (2007).

The parameter of resistance to the change of volume is equal to *K*^*V*^ = 50*pNμm*^−1^. The parameter of resistance to the local change of membrane area is equal to *K*^*Al*^ = 1.67·10^−1^*pNμm*^−1^, while the parameter of resistance to the global change of membrane area is equal to *K*^*At*^ = 1.67*pNμm*^−1^. The parameters that were used to define the Skalak material model of cellular membrane are equal to $k_{s}=0.75\cdot 10^{3}pN{\mu m}^{-1}$ and $k_{\alpha }=75\cdot 10^{3}pN{\mu m}^{-1}$, according to the literature (Skalak et al., 1973). The parameter of resistance to the bending of the membrane is equal to *K*^*B*^ = 10^−1^*pNμm*^−1^.

Simulations using the proposed numerical model were performed for two cases of motion of an individual RBC in the caudal vein plexus of a living zebrafish whose circulation was observed 32 h post fertilization, for an overall time period of 11 s. The geometry of the fluid domain is created directly from the microscopic experimental images. The width of the vein plexus is equal to 100μ*m*. The bounding walls of the fluid domain (the vessel walls) and the intussusceptive pillars are modeled using the Bounce-back approach (Ginzbourg and d'Humières, 1996; Gallivan et al., 1997). This practically means that all particles that collide with the walls, return to the fluid domain with the same velocity, which imposes that the velocity at the walls is equal to zero. In the observed part of the capillary plexus blood is flowing from left to right. The outflow boundary condition is defined at the right boundary wall. This means that normal derivatives at the boundary of the relevant quantities are set to be equal to zero. The left boundary wall of the simulation domain is used to define the inlet velocity of the blood. The velocity profile at the inlet at the beginning of the simulation is defined according to the profile in Poiseuille flow. The value of velocity that is prescribed at nodes that are located on the left boundary of the lattice mesh is calculated using experimental data. The blood flow videos of the capillary plexus region were used to track motion of individual RBCs using imaging techniques. By analyzing the motion of these RBCs, their velocity is calculated and this value is used to define the velocity of the blood flow at the inlet. The maximum value of velocity at the inlet that was used in numerical simulations was equal to 60μ*m/s*.

**Figure 3:**
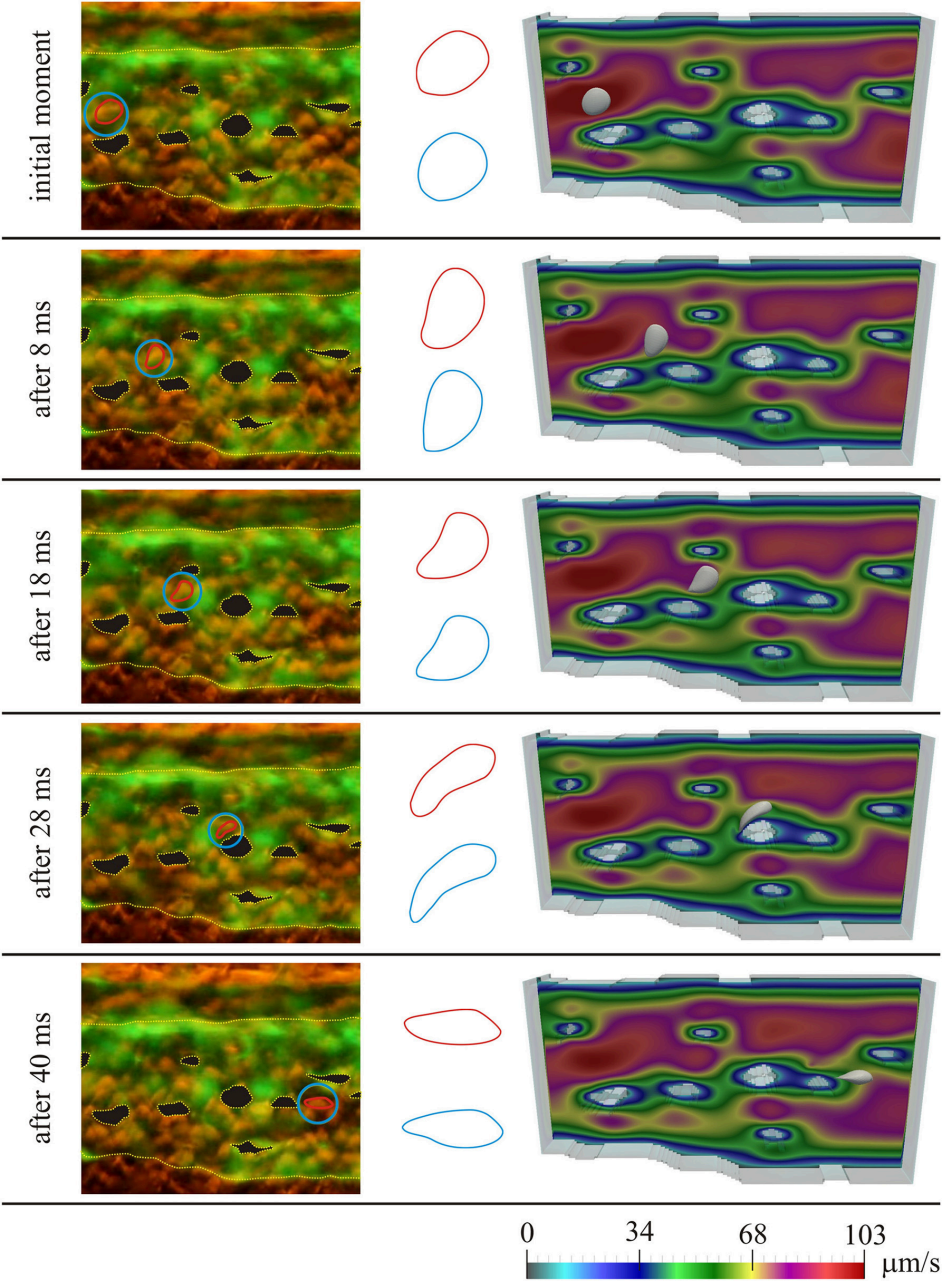
**Comparison of experimental results with results obtained using numerical simulation, for the first initial position of the RBC; left—microscopic image of the zebrafish, with denoted considered RBC; middle—isolated shapes of the considered RBC (red—experiment; blue—simulation); right—results obtained using numerical simulation**. Colors on the images obtained using numerical simulations denote the intensity of the blood velocity, according to the scale bar at the bottom of the Figure. The blood flow through capillary plexus of the living zebrafish was observed 32 h post fertilization.

**Figure 4:**
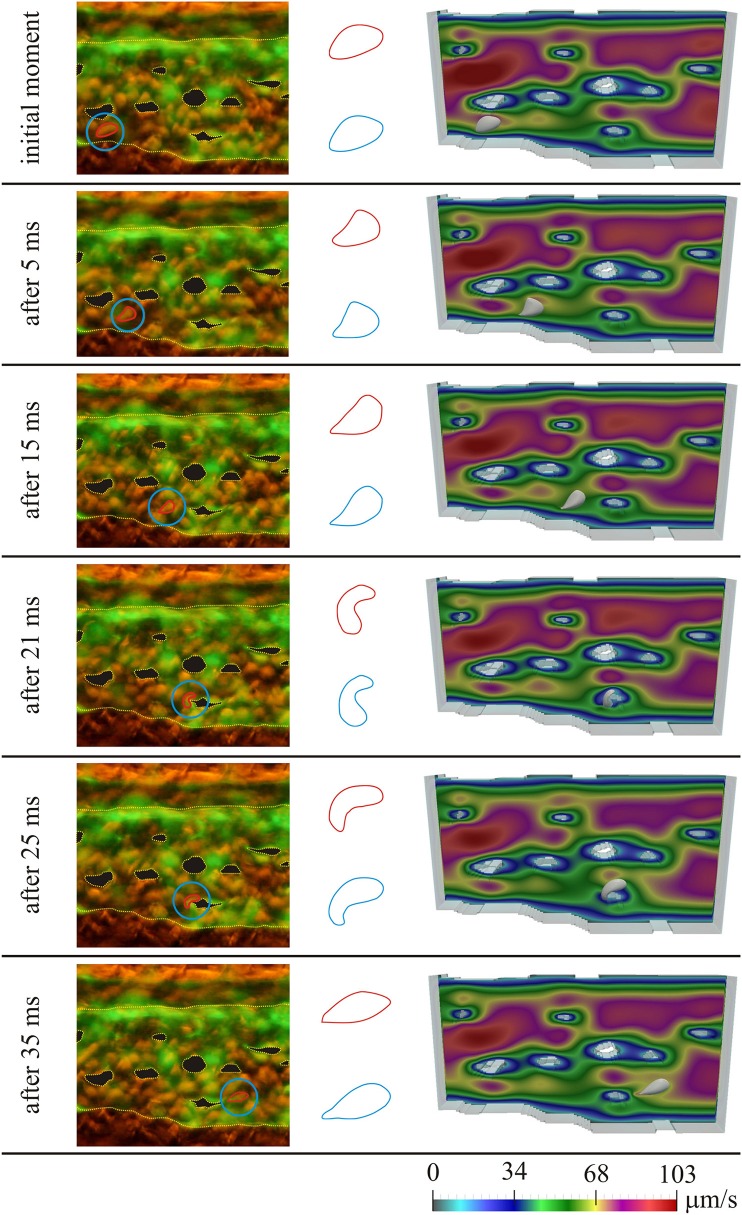
**Comparison of experimental results with results obtained using numerical simulation, for the second initial position of the RBC; left—microscopic image of the zebrafish, with denoted considered RBC; middle—isolated shapes of the considered RBC (red—experiment; blue—simulation); right—results obtained using numerical simulation**. Colors on the images obtained using numerical simulations denote the intensity of the blood velocity, according to the scale bar at the bottom of the Figure. The blood flow through capillary plexus of the living zebrafish was observed 32 h post fertilization.

Figure 3 shows the results for the first considered initial position of the RBC, for several moments in time. On the left the microscopic images of the caudal vein plexus are shown, whereas the considered RBC is denoted by a red line, highlighted in a blue circle. Yellow dotted lines mark the boundaries of the vessel and the pillars within the vessel. On the right the results obtained using numerical simulations are shown. In the middle only the shapes of the considered RBC are isolated for easier comparison (the shape obtained in numerical simulation is denoted by blue color and the shape obtained from the microscopic image is denoted by red color). The case of motion of the RBC that is shown in Figure 3, exhibits the attachment of the RBC to the vascular wall and its subsequent rolling. Figure 4 shows the comparison of results, for the second considered initial position of the RBC, for several moments in time. In this case, the bending and hanging of the RBC on the vascular pillars as well as flowing away and restoration of the original form of the RBC are shown. The shapes obtained in experiments and numerical simulations are also quantitatively compared. The values of area of the cross-section of the RBC along the plane that is parallel to the direction of motion and that contains the center of gravity of the RBC are compared. Table 1 shows the percentage error, that was obtained in numerical simulations, compared to the values that were calculated using experimental data, for the first initial position of the RBC. Table 2 shows the percentage error of the calculated areas of the cross-section of the RBC that was obtained in numerical simulations, compared to the values that were calculated using experimental data, for the second initial position of the RBC. As it can be seen from the isolated shapes of the considered RBCs and the values of errors given in Tables 1, 2, the results obtained using numerical simulation agree well with experimental results.

**Table 1:** **Comparison of experimental results with results obtained using numerical simulation; the percentage error of the area of the cross-section of the RBC obtained in numerical simulation, compared to the value of area calculated from experimental data, for the first initial position of the RBC**.

**Time point**	**Percentage error**
Initial moment	1.741
After 8 ms	−1.882
After 18 ms	2.801
After 28 ms	−2.012
After 40 ms	5.405

**Table 2:** **Comparison of experimental results with results obtained using numerical simulation; the percentage error of the area of the cross-section of the RBC obtained in numerical simulation, compared to the value of area calculated from experimental data, for the second initial position of the RBC**.

**Time point**	**Percentage error**
initial moment	4.264
After 5 ms	1.835
After 15 ms	−4.016
After 21 ms	0.000
After 25 ms	7.288
After 35 ms	5.694

## Discussion and conclusion

Several approaches that deal with the numerical modeling of the behavior of RBCs in the fluid domain are presented in the literature. Pozrikidis (1995) applied the Boundary Element Method (BEM) to model the motion of RBCs. This method was also used by other authors (Kraus et al., 1996; Lac et al., 2004). Ramanujan and Pozrikidis (1998) extended this approach and considered various material models to define the relationship between the reaction force and deformation of the particle. Eggleton and Popel (1998) used the Immersed Boundary Method (IBM) to model the influence of solid on fluid and vice versa. Sui et al. (2008) improved this approach by adding the refinement of the mesh, in order to be able to model the motion more precisely, with higher mesh density near the particle. Discrete particle methods have also been applied to model this type of phenomena. Boryczko et al. (2003) modeled the solid using classic continuum mechanics, while the fluid was modeled as a cluster of particles. On the other hand, based on research published in the literature (Koshizuka et al., 1995), Tsubota et al. (2006) modeled both fluid and solid as a cluster of particles, analyzed their mutual interactions and subsequently modeled the motion of RBCs through the blood plasma. Simulations of a larger number of RBCs and their mutual interactions have been analyzed in several papers (Liu et al., 2004; Dupin et al., 2007; Doddi and Bagchi, 2009). However, in most of the mentioned papers, only the motion of particles and erythrocytes in simpler geometrical conditions has been considered. Under these conditions the feature of erythrocytes to drastically change its shape does not come to the fore. The goal of this paper was to present a model that simulates the motion of RBCs through complex geometrical domains, more precisely through real physical domains obtained experimentally by recording the blood flow through the caudal vein plexus of living zebrafish.

Two initial positions of the RBC within the caudal vein plexus were analyzed. The shapes of the RBC during its motion obtained in numerical simulations agree well with shapes that were extracted from experimental data. The standard deviation of numerical values obtained from the experimental values of the area of the observed cross-section of the RBC is equal to 6.91% for the first considered initial position of the RBC. For the second considered initial position, the standard deviation is slightly greater and is equal to 11.1%. As it can be observed from Tables 1, 2, most values of the obtained error were less than 5%. The greatest error was obtained for the last two moments in time, for the second initial position of the RBC (last two rows in Table 2). These higher values are caused by the fact that in the numerical model a relatively simple approach was used to simulate the intussusceptive pillars. Namely, the pillars were treated as boundary walls, and the RBC was bounced-back from the pillars, just like the fluid particles. If a more detailed interaction between the pillar walls and the RBC was implemented, then this error would be additionally reduced. This will be the main direction of future improvements of the numerical model.

The modeling of oxygen transport and metabolism in organs is significant for the understanding of the functioning of organs and cellular functions from the physiological aspect and the understanding of ischemic and hypoxic conditions. The process of oxygen transport consists of several stages: passage of oxygen molecules through the membrane of the RBC, motion of oxygen together with the RBC through the blood plasma, passage through the vascular wall and arrival to the mitochondria. As stated in the literature, the delivery of oxygen to the tissue is determined mainly by three factors: capillary blood flow, hematocrit, and arterial pressure of oxygen (Li et al., 1997; Dash and Bassingthwaighte, 2006). However, oxygen usage on a cellular or tissue level cannot be measured directly. In order to quantitatively explain phenomena that occur during oxygen transport, it is necessary to use mathematical models and numerical simulations, which have been quite successful in the study of many complex biological systems (Wolkenhauer, 2013).

There have been several methods proposed in literature that mathematically describe the transport of oxygen. Dash and Bassingthwaighte (2006) concluded in their study that a significant decrease in blood flow can cause acute hypoxia and prevent cells from functioning normally. Beyer et al. (2002) proposed a convection-diffusion-reaction mathematical model that simulates the transport of oxygen from RBCs to mitochondria, where they treated RBCs and blood plasma as two separate flows. Li et al. (1997) analyzed oxygen transport at the regional level with imaging techniques using tracer ^15^O-oxygen for positron emission tomography. They observed that with a decrease of capillary blood flow, oxygen delivery by flow to the tissue can become inadequate. Also, when they analyzed tracer kinetics, the initial statement was confirmed—when the capillary blood flow or hematocrit or arterial pressure of oxygen were reduced, the retention of tracer oxygen was prolonged, and the fraction of tracer oxygen in the outflow of the observed blood vessel was reduced.

A more extensive model of O_2_/CO_2_ transport and exchange in the microcirculation, that covers all aspects and all phenomena that happen during this process has yet to be developed. In all previously mentioned models, blood is assumed to consist of two continuous coexisting phases and RBCs are only one “layer” within the blood. The entire microvessel is most commonly described as a tube, where the RBCs are assumed to be located within the central column, while the surrounding region represents blood plasma. The velocity of RBCs and blood plasma can be different within the observed domain and this way the motion of RBCs relative to the blood plasma is taken into consideration. On the other hand, because oxygen is mostly transported through the body within the RBCs, in the form of oxyhemoglobin, the time taken for the transmitted nonextracted oxygen to arrive to the targeted location mainly depends on the velocity of the RBCs. Thus, the aspect of motion of individual RBCs within the blood vessel has to be considered. The method proposed in this paper can be connected to the previous methods modeling the transport of oxygen and can give valuable insights about the motion of RBCs through complex domains of living microvessels.

The numerical model presented in this paper can also be used to analyze erythroid diseases, where disruption of RBC morphology and mechanics occurs. These diseases include hereditary spherocytosis, hereditary elliptocytosis, sickle cell disease etc. The changes in RBCs in these cases have been studied in the literature (Diez-Silva et al., 2010; Fisseha and Katiyar, 2012; Du et al., 2015) and the presented numerical model can be used in combination with these findings to provide additional insight into the mentioned phenomena. The numerical model presented in this paper is capable of simulating the microcirculation, including the complete simulation of dynamics of motion of individual cells. Most numerical models that have been presented in literature so far are limited to simulations in geometrically simple blood vessels. Due to the accuracy of the obtained solutions that was demonstrated through the comparison of the numerical results with experimental results, the proposed model and the developed software can be used for simulations of the complex motion of viscoelastic bodies, such as RBCs, in capillaries with complex geometry.

## Author contributions

TD conceived the study, selected data for numerical simulations and developed the numerical model; SK perfomed experiments with zebrafish; TD and IS performed numerical simulations; SK and IS participated in study conception and assisted in writing the manuscript; VD and NF participated in the study coordination, gave their important intellectual content, participated in data interpretation and contributed to the revision of the manuscript. All authors have read and approved the final manuscript.

## Funding

This paper is supported by grants from Ministry of Education, Science, and Technological Development of the Republic of Serbia (projects number III41007 and ON174028). This paper is also supported by the Swiss National Science Foundation, SCOPES project (JRP: IZ73Z0_152454/1).

### Conflict of interest statement

The authors declare that the research was conducted in the absence of any commercial or financial relationships that could be construed as a potential conflict of interest.
